# Sick and Poor

**Published:** 1871-02

**Authors:** 


### SICK AND POOR.

Many a man who has not a dollar ahead thinks it a great calamity that he has not the means to enable him to get well, while every physician knows that in multitudes of cases the only obstacle to health in the case of the rich is that they are not obliged to work. There is many a "fair ladie," the occupant of a splendid mansion on Fifth Avenue, who, rustling in costliest silks, taking her daily drives in her splendid equipage, walking on velvet carpets and receiving in her saloons the culture and grace and elegance of the city, is at the same time a martyr to maladies all unsuspected by admiring outsiders; yet thin, and frail, and pallid she exists rather than lives; life is a burden rather than a blessing; all that she lacks to put the rose on her cheek, and make existence a happiness, is the necessity of scrubbing floors at a dollar a day, or washing clothes at fifty cents a dozen, to purchase meat and bread enough to keep body and soul together. Hence it is infinitely easier for a poor man to get well than a rich one. The poor man can turn his hand to anything; the unfortunate rich knows nothing; he can't split a rail, or drive a wedge, or drive a cart, or curry a horse, for if he tried the last, he would be so awkward the poor brute would think it was some animal gouging out his ribs, and would incontinently kick his brains out, if indeed there were any there. Hence it many times happens that the fortune of the poor is their poverty. A poor clergyman, on consulting a physician, after having taken all the medicine he could get within ten miles, and got no better fast, was advised to go to work. Some time after, in relating the improvement of his health and the means employed, he writes, "I am making collections for a company and selling farm implements for the most part, stopping on a farm now and then, and working for my board." He was told that he was a fortunate man; that boarding around gave him a wholesome variety of food; and that collecting bills and selling agricultural implements was an honorable and useful employment; that it kept him exercising a great deal in the open air, and was an admirable means of cultivating the powers of persuasive oratory; that bartering in a country store was the foundation of Patrick Henry's immortality, as it gave him opportunities of meeting the sophistries of the common people, of overcoming their prejudices, and observing what kind of arguments reached common minds; and he who can persuade money out of the pocket of a dilatory debtor, and can make a miser buy what he don't want, will soon be skillful enough to move the masses as Patrick Henry did, and win souls for his hire, besides curing himself of dyspepsia, hobgoblins, and other "varmints." Many invalids and a great variety of ailments could be certainly, permanently, and happily cured, if the unfortunate sufferer only had the moral courage to engage in some out-door employment, requiring but moderate activity, even if it yielded only money enough to pay expenses; but mind, in proportion as the yield was greater, the sooner will the patient get well, for money-making is a medicine more efficient than the most potent pill of the apothecary.

